# 1338. Changes in Volume and Diagnoses in a Pediatric Emergency Department during the COVID-19 Pandemic in Northeast Mexico

**DOI:** 10.1093/ofid/ofad500.1175

**Published:** 2023-11-27

**Authors:** Paulina Blanco Murillo, Oscar Tamez-Rivera, Rommy E Pineda-Magaña, Lucia Escobedo-Berumen, Ricardo J Estrada-Mendizabal, Katheryne P Madrazo-Aguirre, Manuel Ruelas-Martinez, Maria Guadalupe Garcia-Lima

**Affiliations:** Tecnologico de Monterrey, Escuela de Medicina y Ciencias de la Salud, San Pedro Garza Garcia, Nuevo Leon, Mexico; Tecnologico de Monterrey, Escuela de Medicina y Ciencias de la Salud, San Pedro Garza Garcia, Nuevo Leon, Mexico; Tecnologico de Monterrey, Escuela de Medicina y Ciencias de la Salud, San Pedro Garza Garcia, Nuevo Leon, Mexico; Centro Medico ABC, Mexico City, Distrito Federal, Mexico; Tecnologico de Monterrey, Escuela de Medicina y Ciencias de la Salud, San Pedro Garza Garcia, Nuevo Leon, Mexico; Tecnologico de Monterrey, Escuela de Medicina y Ciencias de la Salud, San Pedro Garza Garcia, Nuevo Leon, Mexico; Tecnologico de Monterrey, Escuela de Medicina y Ciencias de la Salud, San Pedro Garza Garcia, Nuevo Leon, Mexico; Instituto Nacional de Perinatologia, San Luis Potosí, San Luis Potosi, Mexico

## Abstract

**Background:**

During the first months of the COVID-19 pandemic, a reduction in the volume of patients who sought care in pediatric emergency departments (ED) was observed. An apparent change in discharge diagnoses was also witnessed. There is scarce evidence about the impact of the pandemic on pediatric emergency departments in Mexico and Latin America.

**Methods:**

We conducted a retrospective observational study at a tertiary care hospital in Mexico. We compared the volume and discharge diagnoses of patients aged 0-16 years admitted to the ED during the COVID-19 pandemic from Dec 2020 to Feb 2021 and during the same 3 months in the previous year (Dec 2019 to Feb 2020). As a secondary objective, we compared the subjects’ demographic characteristics and comorbidities. Chi-square test was performed to compare proportions and Student’s t test to compare the median age of patients between the two periods.

**Results:**

In the pre-pandemic period (Dec 2019 to Feb 2020) there were 1,686 admissions to the ED, compared to 941 during the pandemic period (Dec 2020 to Feb 2021). This represented a 44.1% decrease in pediatric admissions to the ED. Median age in months was higher during the pandemic period (50.8) compared to the previous year (69.8) (Table 1). There was a significant increase in the percentage of patients who had a positive history of comorbidities. The percentage of infectious and respiratory diseases significantly declined during the pandemic period, particularly pneumonia (13.1% vs. 1.9%), bronchiolitis (10.7% vs. 1.3%), and rhinopharyngitis (4.1% vs. 1.3%) (Table 2). There was also an increase in intoxication cases (1.7% vs. 3.4%) and accidents (6.6% vs. 14.7%). The percentage of neonatal, surgical, neurological, rheumatological, and gastrointestinal diseases also increased during the pandemic period.

**Figure:**
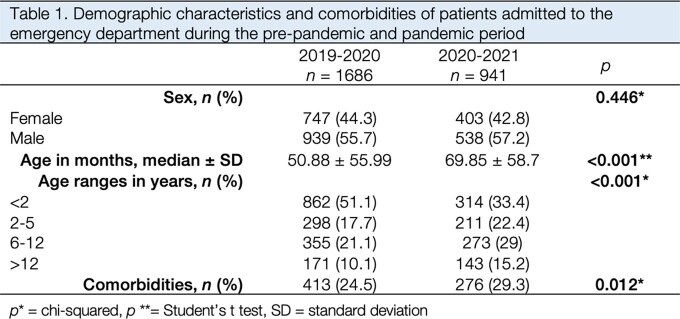

**Figure:**
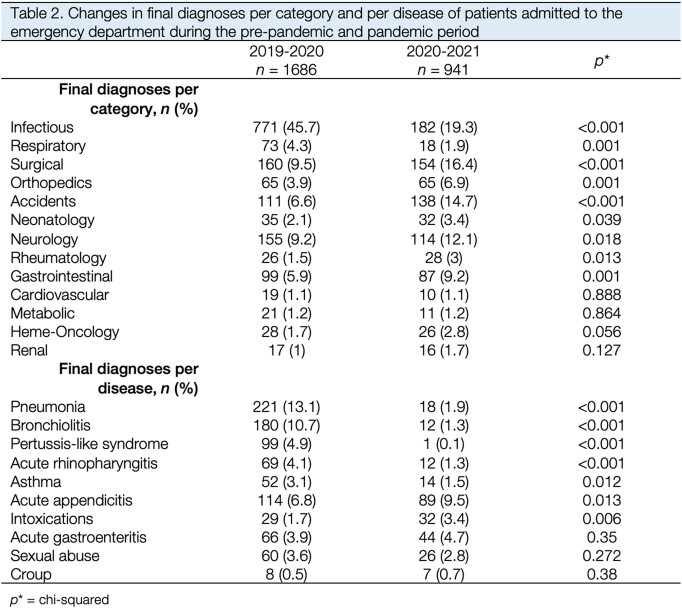

**Conclusion:**

There was a substantial decrease in the total volume of patients as well as a significant change in discharge diagnoses between the pre-pandemic and pandemic studied periods. This study adds to the scarcity of literature available about the impact of COVID-19 on pediatric EDs in Mexico and Latin America. Further studies regarding medical attention-seeking behavior during the pandemic are crucial to planning for the allocation of health resources in case of future health emergencies.

**Disclosures:**

**All Authors**: No reported disclosures

